# Editorial: Current Issues in Nostalgia Research

**DOI:** 10.3389/fpsyg.2021.713534

**Published:** 2021-07-16

**Authors:** Georgios Abakoumkin, Jeffrey D. Green

**Affiliations:** ^1^Laboratory of Psychology, Department of Early Childhood Education, University of Thessaly, Volos, Greece; ^2^Department of Psychology, Virginia Commonwealth University, Richmond, VA, United States

**Keywords:** nostalgia, self, emotion, identity, well-being

In the beginning of the overview text to this Research Topic, we defined nostalgia as “a sentimental longing … for the past” (Pearsall, 1998, p. 1,266) and reported the results of a search in Web of Science for the topic “nostalgia” (in September 2019). Based on the sharp growth over the last five decades, we concluded that the field of nostalgia research is growing exponentially, from 114 publication records in the first decade (1970–1979) to 2,852 publications in the most recent decade (2010–2019), which at that time (September 2019) was not yet concluded. A more recent search (May 2021) revealed 488 relevant publications in 2019, a number that grew to 521 in 2020. This burgeoning interest in nostalgia research is also reflected in the contributions to our Research Topic, which resulted in 12 published articles.

In the mentioned overview, we attributed this development of interest in nostalgia mainly to nostalgia's benefits for individuals and groups. Nostalgia has been identified as a resource that both protects and promotes the self (Sedikides et al., 2015). However, the generality of this view has been challenged (Newman et al., 2020). In our call for this Research Topic, we explicitly advocated for nostalgia research that adds to the existing body of evidence as well as for research on existing controversies. We classify seven out of the 12 contributions as investigating nostalgia's benefits or downsides (Abeyta et al.; Newman and Sachs; Salmon and Wohl; Kersten et al.; Batcho; Rogers; Zhou et al.). Three additional articles are concerned with nostalgia's sociality and its benefits or downsides at the collective level (Abakoumkin et al.;  Green et al.; Behler et al.). Finally, two papers extended nostalgia theorizing into new areas (FioRito and Routledge; Allison and Green). We will now introduce these articles according to this classification, followed by our outlook for possible future avenues for nostalgia research.

## Nostalgia: a Resource or a Challenge?

The first two articles are concerned with the relationship between nostalgia and loneliness. Feelings of loneliness are highly distressing and can even be associated with suicidal ideation (Stroebe et al., 2005). Nostalgia may operate as a resource against loneliness (Zhou et al., 2008). These authors found that nostalgia increases feelings of social support and thereby remedies social connectedness deficits produced by loneliness. Abeyta et al. went a step further. In four studies, they tested and found that nostalgia counteracts low social confidence and low intentions for social approach, thereby reducing loneliness. But a rather different picture emerges from the longitudinal diary study by Newman and Sachs, who found that their participants felt more nostalgic when feeling lonely, which amplified overall negative feelings.

The next two articles demonstrate salubrious effects of nostalgia that concern motivations for a personal future free from addiction or physical pain. Salmon and Wohl conducted an experiential analysis of individuals with gambling problems, who wrote about their past (vs. future). Those who reported more positive (vs. negative) past experiences indicated that they felt more nostalgic about their past life free of gambling as well as a greater readiness to change their gambling behavior. Kersten et al. extended relevant past work on physical pain (Zhou et al., 2012) and found that chronic pain sufferers who engaged in nostalgic (vs. ordinary) event reflection reported lower pain levels. In a second experiment, they found greater pain tolerance for nostalgizers experiencing pressure-oriented pain.

In a study examining both past and future time perspectives, Batcho examined the associations between personal and anticipatory nostalgia (missing something before it has been lost) with her participants' responses to happy and sad stories. Whereas, personal nostalgia was associated with happiness elicited from happy stories, anticipatory nostalgia was associated with sadness elicited from sad stories. Nevertheless, the link between personal nostalgia and positive affect was consistent with previous research (Sedikides et al., 2015). A refinement of the positive relationship between nostalgia and positive affect is provided by Rogers. In this experiment, the usual higher level of positive affect resulting from nostalgic (vs. control) reverie was replicated. However, when the nostalgia manipulation included recalling a past event from a more distant third person (vs. typical first person) perspective, the difference in positive affect between nostalgia and control condition vanished. Zhou et al. also examined anticipatory nostalgia, investigating its role in marketing communications. In four studies, these researchers found that eliciting anticipatory nostalgia regulated participants' affective responses as a function of the valence of the actual experience. Specifically, affective responses were more positive when anticipating losing a negative (vs. a positive) experience.

## Nostalgia at the Collective Level

Nostalgia can also be experienced as a group-level emotion termed collective nostalgia (Sedikides and Wildschut, 2019). Abakoumkin et al. built on previous research that associated nostalgia proneness (nostalgia at the personality trait level), with sociality. Their two studies revealed a more general and direct link between nostalgia proneness and the collective self. In a more specific domain, Green et al. examined the university nostalgia of alumni. In two studies, they found that university nostalgia increased intentions to engage with the alma mater as well as with fellow alumni (e.g., donate more money, attend reunions), and these intentions were mediated by feelings of belonging.

The article by Behler et al. was concerned with a facet of collective nostalgia associated with contentious intergroup relations. They showed that national nostalgia felt by a sample of US voters was associated with greater threat, racial prejudice and greater support for former President Trump and his populist policies, though *personal* nostalgia was indirectly associated with lower prejudice.

## Theoretical Points

Although nostalgia is concerned with the past, FioRito and Routledge propose the possibly counterintuitive view that nostalgia is a future-oriented experience (a similar argument was put forward by Sedikides and Wildschut, 2016). They base this view on ample research showing that nostalgia, by reflecting on the past, fuels future-oriented motivations. In a more specific theoretical contribution, Allison and Green examine the interrelations between nostalgia and heroism. They propose that nostalgia can promote heroism and heroism can promote nostalgia, while, ultimately, nostalgia can support the acquisition of wisdom.

## Outlook

To present the twelve articles of this Research Topic, we classified them into three categories. Naturally, alternative classifications are possible on different theoretical or empirical dimensions. For example, we could have classified the articles on the emotional signature of nostalgia (from positivity to negativity), time perspective emphasis, or applications (ranging from pain reduction to university donations). Regardless of classifications, some issues are still relatively unsettled or even controversial. For example, does nostalgia ease the effects of loneliness or does it amplify them? Our main suggestion for the integration of past work and the development of future research is to attend to conceptualization issues. For example, we think that the way nostalgia was conceptualized and presented to participants was not the same in Abeyta et al. and Newman and Sachs. Another example: the conceptualization of nostalgia with a view to the future might be related to loss (Batcho; Zhou et al.) or to gain (Salmon and Wohl; FioRito and Routledge). In sum, the state of nostalgia science is healthy and far from settled. Broader and more encompassing conceptual frameworks may be proposed to help guide future work as well as facilitate some resolution of currently controversial issues. Research is accelerating rather than waning, and much new territory has yet to be explored.

## Author Contributions

GA and JG contributed substantially and directly both to the relevant work on the Research Topic as well as to the Editorial. Both authors approved the submitted version.

## Conflict of Interest

The authors declare that the research was conducted in the absence of any commercial or financial relationships that could be construed as a potential conflict of interest.
